# Efficacy of a Low-Cost, Inactivated Whole-Cell Oral Cholera Vaccine: Results from 3 Years of Follow-Up of a Randomized, Controlled Trial

**DOI:** 10.1371/journal.pntd.0001289

**Published:** 2011-10-18

**Authors:** Dipika Sur, Suman Kanungo, Binod Sah, Byomkesh Manna, Mohammad Ali, Allison M. Paisley, Swapan K. Niyogi, Jin Kyung Park, Banawarilal Sarkar, Mahesh K. Puri, Deok Ryun Kim, Jacqueline L. Deen, Jan Holmgren, Rodney Carbis, Raman Rao, Nguyen Thu Van, Seung Hyun Han, Stephen Attridge, Allan Donner, Nirmal K. Ganguly, Sujit K. Bhattacharya, G. Balakrish Nair, John D. Clemens, Anna Lena Lopez

**Affiliations:** 1 National Institute of Cholera and Enteric Diseases, Kolkata, India; 2 International Vaccine Institute, Seoul, Republic of Korea; 3 University of Gothenburg, Gothenburg, Sweden; 4 Shantha Biotechnics, Hyderabad, India; 5 Company for Vaccine and Biologicals, Hanoi, Vietnam; 6 Seoul National University, Seoul, Korea; 7 University of Western Ontario, London, Ontario, Canada; 8 Indian Council of Medical Research, New Delhi, India; Massachusetts General Hospital, United States of America

## Abstract

**Background:**

Killed oral cholera vaccines (OCVs) have been licensed for use in developing countries, but protection conferred by licensed OCVs beyond two years of follow-up has not been demonstrated in randomized, clinical trials.

**Methods/Principal Findings:**

We conducted a cluster-randomized, placebo-controlled trial of a two-dose regimen of a low-cost killed whole cell OCV in residents 1 year of age and older living in 3,933 clusters in Kolkata, India. The primary endpoint was culture-proven *Vibrio cholerae* O1 diarrhea episodes severe enough to require treatment in a health care facility. Of the 66,900 fully dosed individuals (31,932 vaccinees and 34,968 placebo recipients), 38 vaccinees and 128 placebo-recipients developed cholera during three years of follow-up (protective efficacy 66%; one-sided 95%CI lower bound = 53%, p<0.001). Vaccine protection during the third year of follow-up was 65% (one-sided 95%CI lower bound = 44%, p<0.001). Significant protection was evident in the second year of follow-up in children vaccinated at ages 1–4 years and in the third year in older age groups.

**Conclusions/Significance:**

The killed whole-cell OCV conferred significant protection that was evident in the second year of follow-up in young children and was sustained for at least three years in older age groups. Continued follow-up will be important to establish the vaccine's duration of protection.

**Trial Registration:**

ClinicalTrials.gov NCT00289224.

## Author Summary

New-generation vaccines against cholera are given orally, to stimulate intestinal immunity. An internationally available oral cholera vaccine (OCV) consists of killed vibrio whole cells together with the B subunit of cholera toxin, is safe, and protects vaccinated individuals against cholera for two years, but this vaccine has seen limited use due to its high cost. We developed a simpler, inactivated whole-cell only OCV that can be produced inexpensively and might therefore be attractive for use in developing countries, as well as for travelers from industrialized countries. We tested this new OCV in a randomized, controlled field trial that enrolled 69,328 individuals aged one year and older living in urban slums of Kolkata, India. At three years of follow-up after receiving at two-dose regimen of this OCV, the vaccinated population experienced 66% protection against all episodes of cholera occurring during the three years, and 65% protection against episodes occurring during the third year. Significant protection was evident in the second year in children vaccinated at ages 1–4 years and in the third year in persons vaccinated at ages of five years and older. Follow-up of the study population will continue for five years to ascertain the duration of vaccine protection.

## Introduction

Cholera is a major global public health problem, causing both epidemic and endemic disease. Although conventional, injectable cholera vaccines have been abandoned as public health tools, modern oral cholera vaccines (OCVs) have been found to be safe and effective [1]. A recently revised World Health Organization (WHO) position paper expands the potential role of vaccination as a preventive tool against both endemic and epidemic cholera [2].

There are two licensed OCVs currently available: one containing cholera toxin B subunit (BS) and killed cholera whole cells (WC), which is licensed in over 50 countries, and the other containing only killed WC, which is licensed in India and Vietnam [1], [3]. A field trial of BS-WC vaccine in Bangladesh found that a three-dose regimen was safe and conferred high grade (85%) short-term protection against cholera; protection was clearly evident throughout the first two years of follow-up, but markedly declined in the third year [4]. An advantage of the WC-only vaccine is its low cost, now at $1.85 per dose to the public sector. We conducted a placebo-controlled, randomized trial to assess the safety and protection conferred by a two-dose regimen of the WC-only vaccine against cholera severe enough to warrant solicitation of medical care. An initial analysis of an ongoing field trial in Kolkata of the WC-only vaccine found a two-dose regimen to be safe and to confer 67% protective efficacy against cholera at two years of follow-up [5]. Here we present results from the third year of follow-up of the Kolkata trial.

## Methods

Details on the study site, study agents, study procedures, and assembly of subjects for this parallel, randomized trial were previously reported [5].The study was performed in a cholera-endemic area in the slums of Kolkata, encompassing a population of ∼109,000. Residents who were at least one year of age and were not pregnant were eligible to participate in the study.

### Interventions and Allocation

Each dose of the killed WC OCV (Shanchol™, Shantha Biotechnics), contains inactivated *Vibrio cholerae* 01 cells representing the El Tor and classical biotypes and the Inaba and Ogawa serotypes, as well as serogroup 0139 cells. Vials containing identical–appearing heat-killed *Escherichia coli* K12 cells were used as placebo. Single-dose vials were labeled with one of four letter codes, two for vaccine and two for placebo. Project staff and study subjects were unaware of the identities of the codes. Participants were randomly assigned, by residential dwelling, to vaccine or placebo groups. Randomization was done before enrollment by an independent statistician (AD), using a random number table. Dwellings were randomized in blocks of 4, corresponding to the 4 code letters used to label vaccine and placebo, within strata defined by the ward of residence and the number of residents in the dwelling (six strata). Each agent was given as a two-dose regimen with an inter-dose interval of at least 14 days. Enrollment and administration of the pre-assigned agents was performed by dosing teams in vaccination centers serving the population. Codes were kept secretly at Shantha Biotechnics and the International Vaccine Institute by staff who were not involved with the trial. The agents were administered in two rounds in 2006: from July 27 to August 13 and from August 27 to September 10, 2006.

### Study Procedures and Definitions

Surveillance was performed in nine community clinics established for the trial and in two hospitals serving the study population. Study physicians completed structured study forms to obtain pertinent clinical information, and fecal specimens were tested for *V. cholerae* as previously described, including identification of 01 and 0139 serogroups with agglutination tests. Biotype was ascertained for all 01 isolates, and the biotype of the cholera toxin genetically encoded was identified as previously described [5], [6]. Confirmation that the subject had indeed visited the treatment site on the date of the visit was assessed through domiciliary visits for all patients whose samples yielded *V. cholerae* O1 or O139. A diarrheal visit was defined as having, in the 24 hours before presentation: 3 or more loose or liquid stools; or, at least 1 loose or liquid stool with blood; or, if 1–2 or indeterminate number of loose or liquid stools were reported, the patient must have exhibited at least some evidence of dehydration, using WHO criteria [7]. The onset of a diarrheal visit was the day on which the patient first reported loose or liquid stools. Diarrheal visits for which the date of onset was less than or equal to 7 days from the date of discharge for the previous visit were grouped into the same diarrheal episode. The onset of a diarrheal episode was the onset of the first diarrheal visit of the episode. The primary endpoint, a cholera episode, was defined as a diarrheal episode in which no component visit was described as bloody, in which a fecal specimen yielded *V. cholerae* O1, and a domiciliary check confirmed that the subject had indeed visited the treatment center for diarrhea on the recorded date of presentation. Demographic surveillance for migrations and deaths among the study population was maintained during the three years of follow-up, and verbal autopsies were done for identified deaths.

### Analysis

The sample size calculation for the trial was previously reported [5]. Prior to the analysis, data were frozen, and a detailed analytic plan was approved by the data and safety monitoring board. The primary analysis was a per-protocol analysis of vaccine protection among subjects who completely ingested two doses of an agent with the assigned treatment code, and included first cholera episodes with onsets between 14 days and 1,095 days after receipt of the second dose. A modified intention-to-treat analysis was done for all individuals who received at least one dose of an agent regardless of the amount ingested, and regardless of whether the agent received was as assigned. First cholera episodes that began from 1 to 1,095 days after the intake of the first dose were included in this analysis. All analyses were conducted and interpreted prior to unblinding of the codes.

Survival analyses were used to calculate vaccine protective efficacy with measurements of the time to the first episode of cholera, censoring the follow-up of individuals who died or migrated out [8]. Kaplan-Meier curves were constructed for descriptive analyses. We also fitted unadjusted and adjusted Cox proportional hazards regression models, after verifying that the proportionality assumptions were fulfilled for all independent variables [9]–[11]. We estimated the hazard ratios by exponentiating the coefficient for the vaccine variable in these models and calculated the vaccine efficacy (PE) as : (1- hazard ratio)×100%. To estimate P values and confidence intervals (CI) for the hazard ratio, we used the standard errors for the coefficients. Robust sandwich variance estimates were used to account for the design effect of cluster randomization, allowing inferences for vaccine efficacy at the individual level [12].

Variables used for stratified randomization as well as baseline variables that were found to be significantly associated with time to event at p<0.10 in bivariate analyses were candidates as independent variables in the final models assessing vaccine efficacy. To avoid overfitting the models, we used a backward elimination algorithm to select independent variables in addition to the vaccination variable. Vaccine efficacy was evaluated in different subgroups that were defined prior to analyses. Heterogeneity of vaccine protection was assessed in these subgroups by analyzing interaction terms in the models. All P values and confidence intervals(CI's) were calculated as one-sided except for assessing heterogeneity of vaccine efficacy in different subgroups, for which stochastic estimates were two-sided. An interim analysis at 2 years of follow-up, using the Haybittle-Peto rule, set the P value for statistical significance for the primary analysis of PE at P<.01 [13]. Because the three-year analysis was the major objective of the trial, all analyses reported in this paper were evaluated at a threshold of P<.0.05, with corresponding one-sided 95% CIs. All statistical analyses were performed using SAS version 9.1. While the initial plan for surveillance was only for three years, follow-up is ongoing to assess the duration of protection up to five years post-vaccination.

### Ethics and Monitoring

The study protocol was approved by the ethics committee of the National Institute of Cholera and Enteric Diseases, the Health Ministry Screening Committee of India and the International Vaccine Institute Institutional Review Board. Written informed consent was obtained from older residents and from the guardians of residents aged 1 to 17 years of age. Additional written assent was obtained from residents aged 12 to 17 years. An independent data and safety monitoring board reviewed the study protocol, assessed serious adverse events, and approved freezing of data and the analytical plan prior to starting the analysis.

## Results

**Figure 1:**
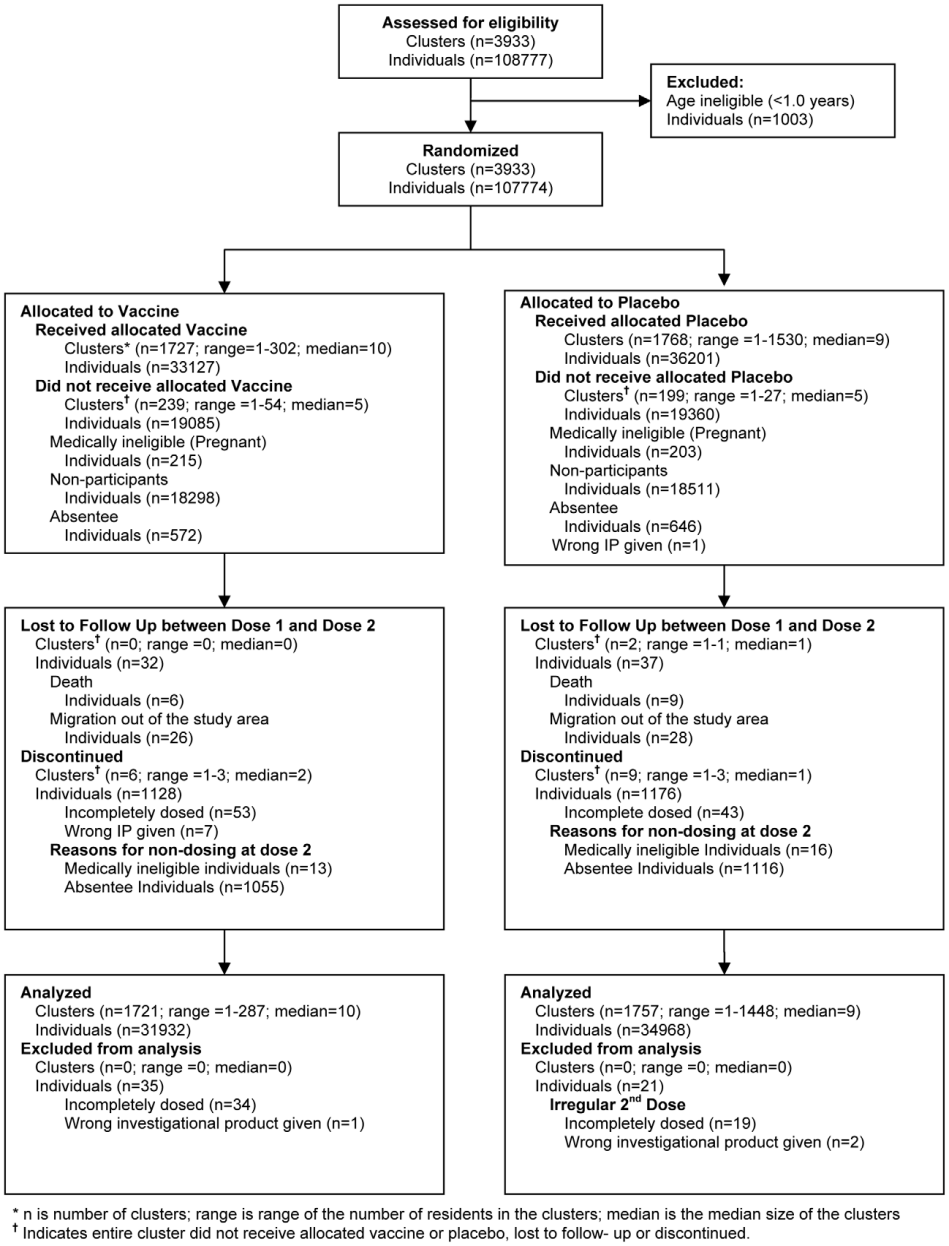
Assembly of subjects for the field trial of killed oral cholera vaccine in Kolkata, India.

The study was prospectively registered at ClinicalTrials.gov (NCT00289224). In the per-protocol analysis, there were 1,721 clusters and 31,932 participants in the vaccine group and 1,757 clusters and 34,968 participants in the placebo group (Figure 1). In the intention-to-treat analysis, there were 1,727 clusters and 33,127 participants in the vaccine group and 1,768 clusters and 36,202 participants in the placebo group. 4,252 and 4,661 participants in the vaccine and placebo groups, respectively, died or migrated out of the study area after the second dose. As previously reported, individual-level and cluster-level baseline characteristics were similar for vaccinees and placebo recipients [5]. There were no substantive imbalances in baseline variables among participants in each arm who were excluded or lost to follow-up.

**Figure 2:**
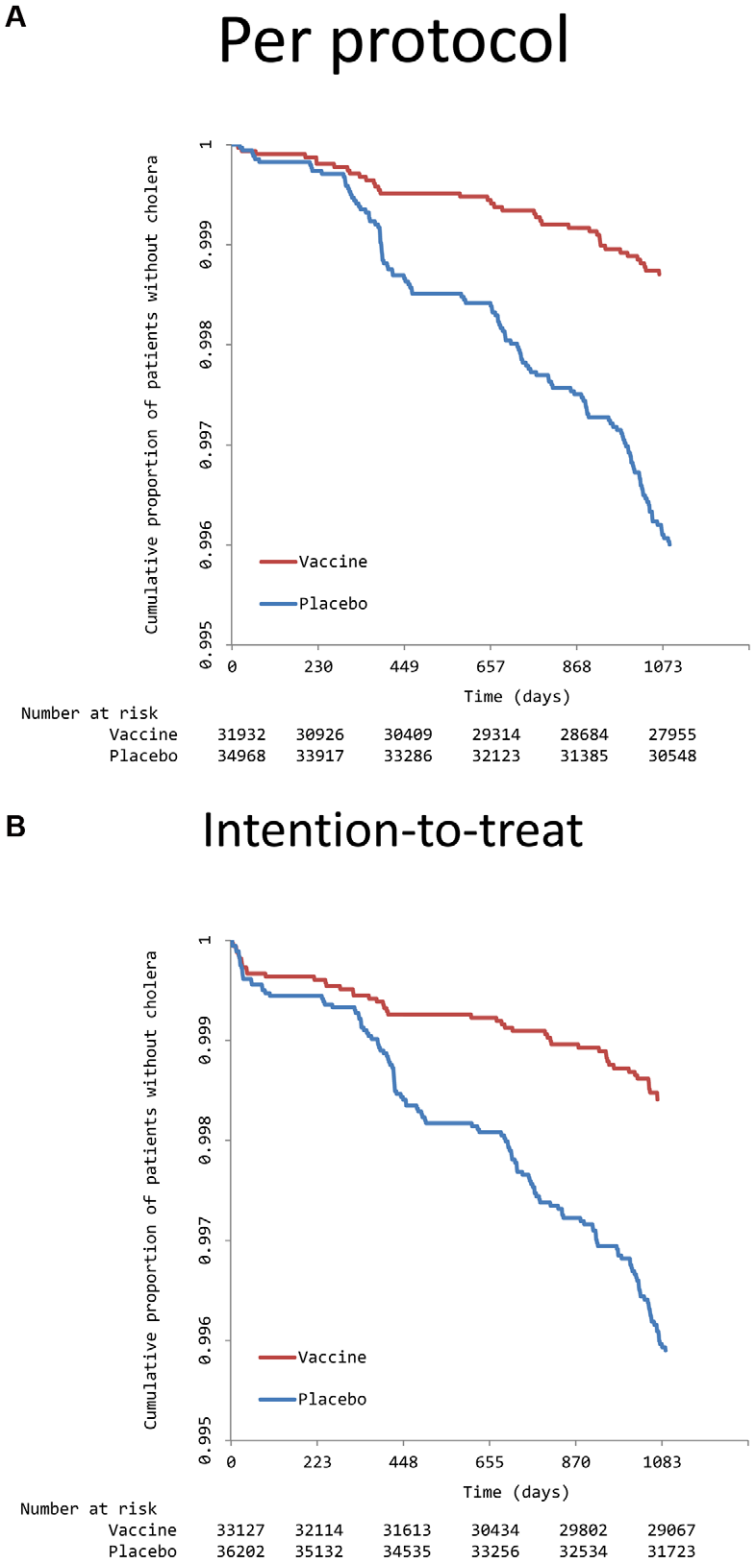
Kaplan-Meier survival curves for the time to first episode of cholera in the per protocol (top) and intention-to-treat (bottom) analyses of the field trial of the killed WC OCV tested in Kolkata.

All detected cholera episodes were due to *V. cholerae* 01, El Tor biotype that genetically encoded classical biotype cholera toxin. As shown in Table 1, 38 and 128 episodes were detected in per-protocol analysis of the vaccine and placebo groups, respectively (adjusted cumulative protective efficacy 66%, one-sided 95% CI-lower bound = 53%, p<0.001). In the intention-to-treat analysis, there were 49 and 137 cholera episodes in the vaccine and placebo groups (adjusted cumulative PE = 61%, one-sided 95% CI lower bound = 47%, p<0.001). Survival curves for each analysis are presented in Figure 2. Most of the isolates were Ogawa serotype. In the per-protocol analysis of protective efficacy against Ogawa cholera, there were 34 and 118 episodes, respectively, in the vaccine and placebo groups (adjusted PE = 68%, one-sided 95% CI lower bound = 54%, p = <0.001). Inaba serotype was detected in only 4 and 10 episodes in the vaccine and placebo groups, respectively (unadjusted PE = 56%, one-sided 95% CI lower bound = −14%, p = .08). There were no deaths due to cholera identified in the the treatment centers, or due to acute watery diarrhea in verbal autopsies among study participants.

**Table 1:** Per protocol analysis of the occurrence of cholera in recipients of the killed WC OCV or K12 *Escherichia coli* placebo by age and year of follow-up after the second dose in Kolkata.

	Age Group	WC	K12	Adjusted PE; 95% LB; P value*(Unadjusted PE; 95% LB; P value)
Year 1	<5 yrs N	2,082+	2,263	17%; −83%; 0.35
	Episodes	8 (3.95)‡	10 (4.55)	(13%; −89%; 0.38)
	5–14 yrs N	7,023	7,698	81%; −12; 0.06
	Episodes	1 (0.15)	6 (0.80)	(82%; −8%; 0.06)
	15+ yrs N	22,827	25,007	66%; −25%; 0.09
	Episodes	2 (0.09)	7 (0.29)	(69%; −16%; 0.07)
	Total N	31,932	34,968	45%; −5%; 0.07
	Episodes	11 (0.35)	23 (0.68)	(48%; 1%; 0.04)
Year 2	<5 yrs N	1,993	2,154	81%; 33%; 0.02
	Episodes	2 (1.03)	13 (6.24)	(83%; 43%; 0.009)
	5–14 yrs N	6,743	7,451	92%; 54%; 0.009
	Episodes	1 (0.15)	13 (1.79)	(92%; 53%; 0.009)
	15+ yrs N	21,796	23,861	62%; 7%; 0.04
	Episodes	6 (0.28)	19 (0.82)	(65%; 17%; 0.02)
	Total N	30,532	33,466	77%; 55%; <.001
	Episodes	9 (0.30)	45 (1.39)	(78%; 57%; <.001)
Year 3	<5 yrs N	1,895	2,037	37%; −47%; 0.18
	Episodes	6 (3.23)	9 (4.51)	(28%; −70%; 0.26)
	5–14 yrs N	6,458	7,098	89%; 62%; 0.002
	Episodes	2 (0.32)	22 (3.16)	(90%; 66%; <.001)
	15+ yrs N	20,623	22,542	64%; 32%; 0.004
	Episodes	10 (0.50)	29 (1.32)	(62%; 31%; 0.004)
	Total N	28,976	31,677	65%; 44%; <.001
	Episodes	18 (0.64)	60 (1.94)	(67%; 47%; <.001)
Total	<5 yrs N	2,082	2,263	43%; 7%; 0.03
	Episodes	16 (2.75)	32 (5.10)	(46%; 12%; 0.02)
	5–14 yrs N	7,023	7,698	88%; 71%; <.001
	Episodes	4 (0.20)	41 (1.89)	(89%; 75%; <.001)
	15+ yrs N	22,827	25,007	61%; 37%; <.001
	Episodes	18 (0.28)	55 (0.79)	(64%; 43%; <.001)
	Total N	31,932	34,968	66%; 53%; <.001
	Episodes	38 (0.43)	128 (1.31)	(68%; 55%; <.001)

*: Protective efficacy (PE), one-sided lower boundary of 95% confidence interval (CI), and one-sided P value for PE.

**+:** Number at risk at onset of cited follow-up period.

**‡:** Number and (rate per 1,000-person years) of cholera episodes in cited group.

Table 1 presents the per protocol analysis by year of follow-up and by age at vaccination. Cumulative three-year vaccine efficacy was highest for children vaccinated at ages 5–14 years (adjusted PE 88%, one-sided 95% CI lower bound = 71%, P<.001), intermediate for persons vaccinated at older ages (61%, one-sided 95% CI lower bound = 37%, P<.001), and lowest for children vaccinated at ages 1–4 years (adjusted PE 43%, one-sided 95% lower bound = 7%, P = .03), and differed significantly (P = .02, two-sided) among the three age groups. Protection of all age groups was 65% during the third year of follow-up (one-sided 95% CI lower boundary = 44%, P<.001), and showed no evidence of decline over time (P = .24, two-sided, for comparison of PE in years 1, 2, and 3 of follow-up). Variations in vaccine protection for each age group, by year of follow-up, did not reach statistical significance (two-tailed P values for comparison of PE in years 1,2, and 3 of follow-up in the 1–4 year, 5–14 year, and ≥15 year age groups were .11, .87, and .98, respectively). Vaccine protection was significant during the third year in the 5–14 year and ≥15 year age groups, but was significant only in the second year in follow-up of younger persons.

## Discussion

Our findings demonstrate that a two-dose regimen of the killed, WC OCV conferred protection of 66% protection during the three years following vaccination. Vaccine protection was clearly evident in the third year of follow-up in persons vaccinated at ages five years and older and during the second year in children vaccinated at 1–4 years of age. Due to small numbers of outcomes during the third year, however, further follow-up will be required to assess the duration of protection in the youngest age group. Protection was clearly evident against El Tor Ogawa, and suggestive against El Tor Inaba, though the latter analysis was limited by a small number of outcome events. Of note, all episodes of cholera were due to *V. cholerae* 01 that manifested the El Tor phenotype but genetically encoded classical biotype cholera toxin, a hybrid strain that now accounts for nearly all cholera cases in many parts of both Africa and Asia and that may be associated with cholera of increased severity [6], [14], [15].

An apparently counterintuitive finding was that vaccine protection was lower in the first year of follow-up than in the subsequent two years. However, the most likely explanation for this finding is chance variation, as there were no significant differences in estimates of vaccine protection, either for all age groups combined or for the <5 year , 5–14 year, and ≥15 year age groups individually, during the three years of follow-up.

Comparing the results of different vaccines tested in different trials provides less conclusive evidence of their comparative efficacy than head-to-head comparisons of vaccines in the same trial. Nevertheless, it is of interest to contrast the long-term results for the killed WC OCV studied in this trial with those for the killed BS-WC OCV tested in three doses in Bangladesh in the 1980s, the only evaluation of BS-WC with long-term follow-up [4]. In contrast to the trial of killed WC OCV in Kolkata, which demonstrated efficacy during the third year of follow-up, BS-WC vaccine's protection against cholera in Bangladesh was significant only during the first two years of follow-up. Of interest, protection by BS-WC vaccine in the Bangladesh trial was also lowest and of shortest duration in the under-five age group, an observation that has been attributed to the lower level of pre-existing, anti-cholera immunity in this group, owing to less exposure to natural cholera infections in the youngest group.

It should be emphasized, however, that long-term protection is only one consideration for the use of an OCV. Enhanced short-term protection may be a distinct advantage when considering the use of a vaccine in self-limited outbreaks of cholera. In this respect, the BS-WC OCV has been shown to confer 85% protection lasting 4–6 months after dosing [16], Another advantage of BS-WC OCV is its ability to confer cross-protection against LT-producing enterotoxigenic *Escherichia coli* diarrhea for several months after dosing [17].

The potential of the killed WC OCV tested in this study for use in control of endemic and epidemic cholera is substantial. However, much remains to be done. The study remains blinded and surveillance will continue to assess the duration of protection provided by the vaccine up to five years after dosing. Increasing access to this vaccine is important, not only in India, where it is currently licensed, but also in other cholera-endemic countries. Access should be increased in the near future by WHO prequalification of the vaccine so that it may be purchased by UN agencies for use in other countries for disease control.

## Supporting Information

Checklist S1 **CONSORT checklist.**
(DOC)

Protocol S1 **Trial protocol.**
(DOC)
