# Supplementary material for: Efficacy of a Low-Cost, Inactivated Whole-Cell Oral Cholera Vaccine: Results from 3 Years of Follow-Up of a Randomized, Controlled Trial
Source: PLoS Negl Trop Dis. 2011 Oct 18;5(10):e1289. doi: 10.1371/journal.pntd.0001289 (PMC3196468; doi:10.1371/journal.pntd.0001289)
Supplement: Protocol S1 — Trial protocol. (DOC) [file pntd.0001289.s002.doc]

**A RANDOMIZED CONTROLLED TRIAL OF THE BIVALENT KILLED WHOLE CELL ORAL CHOLERA VACCINE**

**IN EASTERN KOLKATA, WEST BENGAL, INDIA**

| COLLABORATORS: | Indian Council of Medical Research, Ansari Nagar, New Delhi 110029, India, Telephone (91-11) 2651-7204, Fax (91-11) 2686-8662  National Institute of Cholera and Enteric Diseases, Beliaghata, Kolkata 700010, Eastern Kolkata, West Bengal, India, Telephone (91-33) 2353-7519, Fax (91-33) 2350-5066  Shantha Biotechnics PVT LTD, Serene Chambers, 3rd Floor, Road No. 7, Banjara Hills, Hyderabad 500 034 India, Telephone (91-40) 23543010, Fax (91-40) 23541713  International Vaccine Institute, Seoul National University Campus, Shilim Dong, Kwanka-Ku, Seoul, Korea Telephone (82-2) 872-2801, Fax (82-2) 872-2803 |
| --- | --- |
| Investigational product: | Bivalent (anti-O1, anti-O139), Killed, Whole-cell Oral Cholera Vaccine |
| Control Product: | Killed oral *Escherichia coli* K12 strain placebo |
| Principal Investigator: | Dr Sujit Kumar Bhattacharya, National Institute of Cholera and Enteric Diseases |
| Co-Investigators : | Drs Dipika Sur, Byomkesh Manna, National Institute of Cholera and Enteric Diseases |
| On-site clinical monitor: | Dr Asoke Chowdhury |
| MEDICAL MONITOR: | Dr Raman, Shanta Biotechnics |
| EXTERNAL MONITOR: | Ms Eunhee Shin |
| DATA MANAGEMENT: | Dr Byomkesh Manna, Dr Mohammad Ali, Mr Vinay Kumar Gupta |
| statisticianS: | Dr Byomkesh Manna, Prof Allan Donner |
| Biological laboratory: | Dr Swapan Kumar Niyogi (NICED), Dr Rodney Carbis (IVI),  Prof Jan Holmgren (University of Gothenburg, Sweden) |
| Data Safety Monitoring Board (DSMB): | Drs Roger Glass (CDC, USA), Lt Gen Raghunath, BS Ramakrishna Duncan Steele (WHO, Geneva), Taesun Park (IVI), |
| IVI SCIENTISTS | Drs John D Clemens, Rodney Carbis, Lorenz von Seidlein Jacqueline Deen, Anna Lena Lopez |
| Date: | October 2006 |

**Table of Contents**

|  | **PAGE** |
| --- | --- |
| STUDY SYNOPSIS | 4 |
| ABBREVIATIONS AND DEFINITIONS | 5 |
| 1.INTRODUCTION | 6 |
| 1.1 BACKGROUND | 6 |
| 1.2 STUDY RATIONALE | 7 |
| 2. STUDY OBJECTIVES | 8 |
| 2.1 PRIMARY OBJECTIVES | 8 |
| 2.2 SECONDARY OBJECTIVE | 8 |
| 3. STUDY DESIGN | 8 |
| 3.1 DESCRIPTION OF FIELD SITE | 8 |
| 3.2 ADMINISTRATION, LABORATORY, AND DATA MANAGEMENT FACILITIES | 11 |
| 3.3 TYPE OF STUDY | 11 |
| 3.4 ELIGIBILITY CRITERIA | 11 |
| 3.5 ALLOCATION OF STUDY AGENTS | 12 |
| 3.6 BLINDING AND CODE BREAKING PROCEDURES | 12 |
| 3.7 DURATION OF THE STUDY PERIOD FOR ONE SUBJECT | 12 |
| 4. STUDY PROCEDURES | 12 |
| 4.1 CENSUSES | 13 |
| 4.2 FOLLOW-UP AFTER IMMUNIZATION | 13 |
| 4.2.1 FOLLOW-UP FOR ADVERSE EVENTS | 13 |
| 4.2.2 SEROLOGY | 13 |
| 4.2.3 SURVEILLANCE FOR CHOLERA | 14 |
| 4.3 PREMATURE TERMINATION | 15 |
| 5. ADVERSE EVENTS | 15 |
| 5.1 DEFINITIONS | 15 |
| 5.2 REPORTING OF SERIOUS ADVERSE EVENTS | 15 |
| 6. VACCINE SUPPLIES | 16 |
| 6.1 STUDY AGENTS (VACCINE AND PLACEBO) | 16 |
| 6.2 PACKAGING AND CODING | 17 |
| 6.3 STORAGE CONDITIONS | 17 |
| 6.4 INVESTIGATIONAL PRODUCT ACCOUNTABILITY | 17 |
| 6.5 ADMINISTRATION OF STUDY AGENTS | 17 |
| 6. DATA MANAGEMENT PROCEDURES | 18 |
| 8. STATISTICAL CONSIDERATIONS | 18 |
| 8.1 ENDPOINTS | 18 |
| 8.2 SAMPLE SIZE CALCULATION | 18 |
| 8.3 ANALYSIS PLANS | 19 |
| 8.4 POTENTIAL BIASES | 20 |
| 9. MONITORING, AUDITING, INSPECTION | 20 |
| 9.1 RESPONSIBILITIES OF THE INVESTIGATOR(S) | 20 |
| 9.2 RESPONSIBILITIES OF THE COORDINATOR(S) | 20 |
| 9.3 RESPONSIBILITIES OF THE DSMB | 20 |
| 10. ETHICAL AND REGULATORY STANDARDS | 21 |
| 10.1 ETHICAL PRINCIPLES | 21 |
| 10.2 LAWS AND REGULATIONS | 22 |
| 10.3 INFORMED CONSENT | 22 |
| 10.4 INSTITUTIONAL REVIEW COMMITTEE (IRB) | 22 |
| 11. ADMINISTRATIVE ASPECTS | 22 |
| 11.1 RECORD RETENTION | 22 |
| 11.2 PUBLICATIONS | 22 |
| 11.3 PROTOCOL AMENDMENTS | 23 |
| 12. REFERENCES | 24 |
| APPENDIX 1. INVESTIGATOR’S STATEMENT | 26 |
| APPENDIX 2. INFORMED CONSENT AND ASSENT FORMS | 27 |
| APPENDIX 3. VACCINATION RECORD |  |
| APPENDIX 4. ADVERSE EVENT FORMS |  |
| APPENDIX 5. BLOOD COLLECTION FORM |  |
| APPENDIX 6. SURVEILLANCE FORMS |  |
| CASE REPORT FORM (CRF) – DAY 0 |  |
| LABORATORY TRANSMITTAL / RESULT FORM |  |
| CHOLERA FOLLOW-UP FORM |  |
| VERBAL AUTOPSY FORM |  |
| APPENDIX 7. ORGANOGRAM |  |
| APPENDIX 8. BUDGET |  |
| APPENDIX 9. CURRICULUM VITAE OF INVESTIGATORS |  |

**STUDY SYNOPSIS**

| **Study type** | Clinical Trial (phase I phase II phase III phase IV) |
| --- | --- |
| **Objectives** | Primary objective:  To estimate the efficacy of a two-dose primary regimen of the oral killed bivalent cholera vaccine when administered to residents at least 1 year of age of eastern Kolkata, West Bengal, India, in preventing culture-proven *V. cholerae* O1 diarrhoea episodes severe enough to require treatment in a health care facility.  Secondary objectives:   1. To estimate the efficacy of the vaccine in preventing:  - Culture-proven *V. cholerae* O1diarrhoea episodes associated with severe dehydration; - Episodes of acute watery diarrhoea associated with severe dehydration; - Episodes of acute watery diarrhoea severe enough to require treatment in a health care facility; and - All the above endpoints stratified by age (less than 5 and over 5 years)  1. To evaluate whether the vaccine induces acceptable serum vibriocidal responses in relation to the placebo group. |
| **Design** | Randomized, double-blind, placebo-controlled trial |
| **Subjects** | All enumerated healthy consenting subjects, residing in Wards 29, 30 and 33 Eastern Kolkata, West Bengal, India. Subjects will be included if they are permanent residents of the study site, not pregnant, and at least 1 year of age during vaccination. |
| **Sample size** | 110,000 subjects in the study area |
| **Agents** | Bivalent, killed, whole-cell oral cholera vaccine and placebo |
| **End-points** | a. Efficacy will be assessed by comparing the following between the vaccine and control groups:   - Culture-proven *V. cholerae* O1 diarrhoea episodes severe enough to require treatment in a health care facility (primary endpoint); - Culture-proven *V. cholerae* O1diarrhoea episodes associated with severe dehydration; - Episodes of acute watery diarrhoea associated with severe dehydration; - Episodes of acute watery diarrhoea severe enough to require treatment in a health care facility; and - All the above endpoints stratified by age (less than 5 and over 5 years)   b. Immunogenicity will be assesses by comparing serum vibriocidal responses in the vaccine and control groups. |
| **Study period** | 4 years:   - 8 months for preparation, census, and mass vaccination - 36 months follow-up over 3 cholera seasons (*We may extend to additional months of follow-up depending on availability of funds*.) - 6 months for close-out census, data cleaning and analysis |

**ABBREVIATIONS AND DEFINITIONS**

##### CHW – Community Health Worker

##### CRF – Case Report Form

DSMB – Data Safety Monitoring Board

IDH – Infectious Disease Hospital (Kolkata)

GMP - Good Manufacturing Practices

##### IVI – International Vaccine Institute (Seoul)

ICMR – Indian Council of Medical Research (New Delhi)

IRB – Institutional Review Board

##### NICED – National Institute of Cholera and Enteric Diseases (Kolkata)

NIHE – National Institute of Hygiene and Epidemiology (Hanoi)

VABIOTECH - Company for Vaccine and Biological Production No. 1 (Hanoi)

SAE – Serious Adverse Event

SHANTA – Shantha Biotechnics Pvt Ltd (Hyderabad)

*V. cholerae* – *Vibrio cholerae*

WHO – World Health Organization (Geneva)

**1. INTRODUCTION**

1.1 BACKGROUND

Cholera remains a serious public health problem worldwide. In 2002, a total of 142,311 cases and 4, 564 deaths were reported to the World Health Organization (WHO) from 52 countries primarily in Africa, Asia and Latin America. The true figures are likely to be much higher due to underreporting. Besides the high mortality and morbidity figures, cholera outbreaks cause economic and social disruption as well.

In India, cholera is a persistent problem particularly in States such as West Bengal, Maharastra, Andra Pradesh, Tamil Nadu, Karnataka, Delhi, and Kerala (1). Several districts in the country report explosive outbreaks of cholera (2-4). Since the dramatic appearance of the new serotype O139 Bengal in 1992 in Madras (5), both *V cholerae* O1 and O139 are present in India.

Provision of safe water and food, establishment of adequate sanitation, and implementation of personal and community hygiene constitute the main public health interventions against cholera. These measures cannot be implemented fully in the near future in most cholera-endemic areas. A safe, effective, and affordable vaccine would be a useful tool for cholera prevention and control.

A parenterally administered killed whole cell cholera vaccine was widely available for many years. However, this vaccine offered at best only about 40-50% protection for about 3 to 6 months and produced unpleasant side effects in many vaccinees. In view of these limitations, the vaccine has not been considered satisfactory for general public health use and in 1973 the 26^th^ World Health Assembly abolished the requirement in the International Health Regulations for a certificate of immunization against cholera (6).

Considerable progress has been made during the last decade in the development of new generation oral vaccines against cholera. These have already been licensed in some countries and are now being considered for wider public health application. The World Health Organization recently recommended that the newer generation cholera vaccines be considered in certain endemic and epidemic situations, but indicated that demonstration projects are needed to provide more information about the costs, feasibility, and impact of using these vaccines (7).

A killed oral cholera vaccine was developed by Prof Jan Holmgren in Sweden and consists of inactivated whole cells of *V cholerae* O1 with a purified recombinant–DNA derived B-subunit of the cholera toxin. Large scale field trials of the vaccine in Bangladesh and Peru showed that both the killed whole cell vaccine containing the B-subunit, as well as the killed whole cell preparation alone, conferred significant protection for recipients for up to 3-5 years depending on age. An initial protection of 85-90% was obtained with the killed vaccine containing the B-subunit but this level of protection declined to about 50% after 3 years. The oral vaccine lacking the B-subunit gave a somewhat lower initial level of protection but after 6 months the protection afforded by the two vaccines was similar (8, 9). The vaccine is licensed in several industrialized countries and is used mainly by Western travellers. Unfortunately, the vaccine is prohibitively expensive for public health use in developing countries.

Starting in the mid-1980s, following technology transfer from Prof Jan Holmgren, Vietnamese scientists at the National Institute of Hygiene and Epidemiology (NIHE) in Hanoi developed and produced an oral, killed cholera vaccine for the country’s public health programs (10). A two-dose regimen of a first generation monovalent (anti-O1) cholera vaccine produced at US$ 0.10 per dose underwent a field trial in Hue, Vietnam (11). The study was not formally randomised: the vaccine was assigned on the basis of a systematic allocation scheme and the control group did not receive a placebo. The calculated efficacy against El Tor cholera was 66% in fully immunized adults and children. Protection against non-cholera was assessed and none was found suggesting a non-biased study design. Subsequently, killed 0139 whole cells were added to the Vietnamese vaccine due to the emergence of the new form of epidemic cholera caused by this serogroup. A study found the bivalent vaccine to be safe and immunogenic in adults and children one year and older (12).

Due to the limitations of the Hue study and the addition of a new component to the vaccine, a large-scale field trial of the Vietnamese bivalent (O1 and O139) killed, oral cholera vaccine in non-pregnant persons 1 year and older was conducted in Nha Trang, Vietnam (unpublished data, Clemens J, et al.). Approximately 340,000 persons (20% between 1 to 3 years of age) were randomised to receive a two dose regimen of the vaccine or placebo. No *V cholerae* was isolated during the surveillance thus effectiveness could not be demonstrated. However, surveillance for potential side-effects showed no difference between the vaccine and placebo groups. The data were reviewed by the Data Safety Monitoring Board convened by the WHO and they concluded that the vaccine is safe.

The Vietnamese vaccine has several distinct advantages over the Swedish vaccine. In contrast to the Vietnamese vaccine, the Swedish vaccine is prohibitively expensive for use in developing countries, requires a buffer during administration, and due to its B-subunit component has strict cold chain requirements.

Since licensure of the oral cholera vaccine in Vietnam, more than 5 million doses have been administered without any report of serious adverse events. The vaccine is produced according to recommended guidelines (6) at the NIHE’s newly privatized arm, the Company for Vaccine and Biological Production No. 1 (VABIOTECH) in Hanoi. VABIOTECH is working towards WHO Good Manufacturing Practices (GMP) certification, which they hope to receive in the next few years. The cholera vaccine production technology has been transferred to pharmaceutical production facilities in Indonesia and China. In Vietnam, the vaccine is delivered by the Expanded Programme of Immunization through mass immunization of high risk populations. Recently the vaccine was reformulated in order to comply with WHO standards to further internationalize this vaccine. Phase II trials of this reformulated vaccine have been performed among 148 adults in SonLa,Vietnam (18) and among 100 adults and 100 children in Kolkata, India (19).

The SonLa study revealed that the vaccine is safe and immunogenic among adults (18). No serious adverse events were reported. No statistically significant differences were noted among vaccine and placebo recipients as regards reported adverse events. These events were mostly mild. 90% of vaccinees developed a ≥4 fold rise in vibriocidal antibodies to *V.* *cholerae* O1 whereas none of the placebo recipients did. Serum vibriocidal antibody geometric mean titres (GMT) increased more than 20 fold from baseline among vaccinees compared to no significant increase from baseline among placebo recipients.

Preliminary results from the study in Kolkata (19) revealed that the vaccine is safe among adults and children. There were no significant differences in adverse events reported among the placebo and vaccine groups. Serious adverse events were reported in 1 adult and 3 children, these however were not related to the vaccine. The adult and children were members of a family of 6 who all had vomiting severe enough to require hospitalization for 1 day. Two of the children were placebo recipients and the adult and 1 child were vaccinees. Two other family members who were not study participants also had severe vomiting. There were no significant differences in the baseline serum vibriocidal antibody GMT of adults. Vibriocidal antibody GMT of adults had a significant 4 fold rise from baseline. 53% of vaccinees seroconverted compared to none of the placebo recipients. These results are better than the study in a cholera endemic area in Bangladesh, where 36% of vaccinees and 8% of placebo recipients seroconverted after the second dose (16).

A double-blind randomised phase III trial of the reformulated oral killed bivalent cholera vaccine in an urban slum site in Kolkata is proposed by the National Institute of Cholera and Enteric Diseases (NICED) in collaboration with the International Vaccine Institute (IVI). The IVI has negotiated an agreement between VABIOTECH and Shantha Biotechnics PVT LTD for the vaccine and placebo to be used during the trial. VABIOTECH will produce bulk oral killed bivalent cholera vaccine under quality control conditions to be supervised by IVI staff. Shanta will purchase bulk vaccine from VABIOTECH, fill and finish the bulk vaccine, and obtain regulatory clearance for use of the vaccine in the phase III trial. Shanta will purchase the bulk placebo agent for use in the trial from IVAC (also in Vietnam) and Shanta will fill and finish the placebo and obtain regulatory clearance for use of the placebo in the phase III trial. Shanta will later obtain the technology for future production of the oral killed bivalent cholera vaccine. The results of this trial will pave the way for the use of this vaccine in India and other cholera-endemic areas.

1.2 STUDY RATIONALE

Currently there is no oral cholera vaccine licensed in India. There is an urgent need to demonstrate efficacy of the bivalent oral killed cholera vaccine by conducting a double-blind randomised phase III trial in a cholera-endemic area. The trial would pave the way for the introduction of the vaccine into national immunization programmes in India. The trial would also be important in the internationalization of the vaccine so that the vaccine can be licensed in other countries where it is needed.

**2. STUDY OBJECTIVES**

Primary objective: To estimate the efficacy of a two-dose primary regimen of the oral killed bivalent cholera vaccine when administered to residents at least 1 year of age of eastern Kolkata, West Bengal, India, in preventing culture-proven *V. cholerae* O1 diarrhoea episodes severe enough to require treatment in a health care facility.

Secondary objectives:

1. To estimate the efficacy of the vaccine in preventing:

- Culture-proven *V. cholerae O1* diarrhoea episodes associated with severe dehydration;

- Episodes of acute watery diarrhoea associated with severe dehydration;

- Episodes of acute watery diarrhoea severe enough to require treatment in a health care facility; and

- All the above endpoints stratified by age (less than 5 and over 5 years)

1. To evaluate whether the vaccine induces acceptable serum vibriocidal responses in relation to the placebo group.

**3. STUDY DESIGN**

3.1 DESCRIPTION OF THE FIELD SITE

Kolkata is the third largest city in India and is divided into 141 administrative wards. The study site is an impoverished urban area, encompassing most of Ward 29 and all of Ward 30 as shown below. Households in the study area are densely grouped into premises. Each premise usually has a common latrine and water supply. The population is relatively stable with a household median number of years of residence of 35.

Figure 1. The study site (Wards 29, 30 and 33) among the wards of Kolkata

| 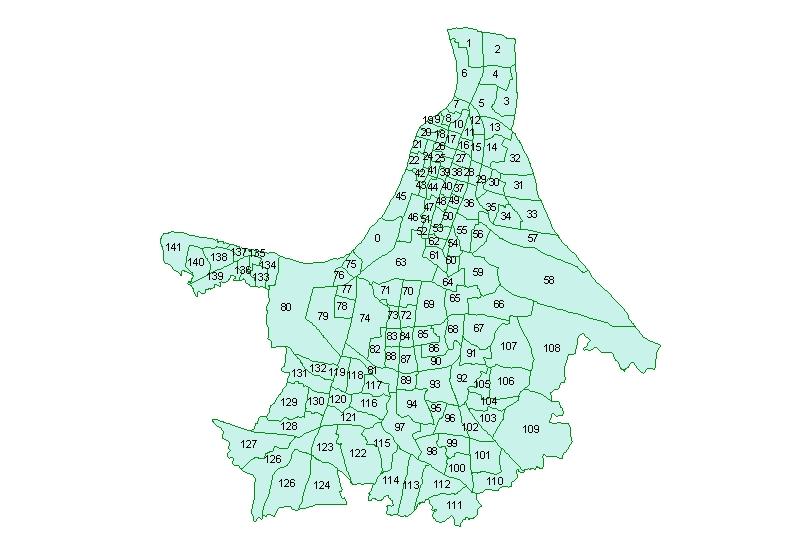 |
| --- |

Preparatory activities have been conducted in the study site (Ward 29 and 30). These include community mobilization starting in late-2002; a baseline census of residents carried-out from January to March 2003; setting-up of 5 health outposts within the wards (approximately one unit for every 10,000 population) and 2 in referral hospitals (Infectious diseases Hospital or IDH and B.C. Roy Children’s Hospital); followed by an on-going health outpost-based passive surveillance of diarrhoea to document the incidence of cholera in the community. ID cards and computerised and printed census booklets are used to identify the individuals and their unique identification number during their presentation to any of the project health outposts. In addition, community health workers (CHWs) visit each individual in the study site once a month to find out about any diarrhoea episodes, hospitalization, or death using bound books pre-printed with the household members’ names. The CHWs encourage consultation for diarrhoea at any of the outposts and inform their field supervisor about hospitalizations and deaths. Hospitalizations are followed-up to check for possible cholera. A verbal autopsy is done for all reported deaths after an appropriate grieving period but within 3 months (see Verbal Autopsy Form, Appendix 6). However, during the period of the mass vaccination, each death detected in the study site will be investigated immediately. Cholera cases are followed-up at home 7 days after presentation to verify identification and record outcome.

In Kolkata, health care is provided by 7 referral government hospitals and various traditional practitioners, private clinics, and private nursing homes. Two of these referral hospitals are included in the health outpost-based passive surveillance of diarrhoea in Wards 29 and 30. IDH, the referral centre for West Bengal for severe diarrhoea cases (including cholera), is in Beliaghata, about 3 kilometres from the study site. B.C. Roy Hospital, the referral centre for severely ill children, is adjacent to the study site.

Using a dynamic cohort as the denominator (based on a baseline and follow-up census one year later), we calculated the incidence of cholera by age-group as shown in the figure below (13). All of the isolates were identified as El Tor Ogawa *Vibrio cholerae* O1. There was no repeat cholera episode in any individual.

**Figure 2: Age-specific incidence per 1,000 person-years of cholera cases**

**1 May 2003 to 30 April 2004 (13)**

We also calculated the incidence of cholera in individuals over 1 year of age (target population for vaccination) in a fixed cohort fashion during the first and second year of surveillance, as shown below:

**Table 1: Incidence of cholera among those over 1 year of age in the study area during the first and second year of surveillance**

| **Time period** | **Denominator** | **Numerator** | **Incidence of cholera / 1000 persons / year** |
| --- | --- | --- | --- |
| May 1, 2003 to April 30, 2004 | Baseline study population over 1 year old = **56,737**  (Age calculation: Oct 31, 2003 as the ref date) | Cholera cases > 1 year of age that link to the baseline population = **107**  (Age calculation: on presentation - CRF-0) | **1.89** |
| May 1, 2004 to April 30, 2005 | Updated population after census-2 over 1 year old = **60,220**  (Age calculation: Oct 31, 2004 as the ref date) | Cholera cases > 1 year of age that link to the updated population = **51**  (Age calculation: on presentation - CRF-0) | **0.85** |

3.2 ADMINISTRATION, LABORATORY AND DATA MANAGEMENT FACILITIES

NICED is a centre of excellence in basic and applied research in the field of diarrhoea and other infectious diseases. It is an organization of the Indian Council of Medical Research (ICMR), which is the premier research institution in India. The multifaceted activities of NICED include pursuit of its research goals in different aspects of diarrhoeal and other infectious diseases, organization of workshops and training programmes for manpower development, assistance to various State Governments in conducting investigations on disease outbreaks, suggestion for control measures and provision of referral services to several laboratories in different parts of the country. The NICED is a WHO-accredited collaborating centre for cholera. As part of the health outpost-based passive surveillance of diarrhoea, a fully-staffed and equipped data management room and laboratory have been set-up.

3.3 TYPE OF STUDY

The study design is a two-arm randomized double-blind placebo-controlled trial. Clusters will serve as the unit of randomization with 1:1 placebo-to-control ratio. Clusters are defined as geographically contiguous units.

3.4 ELIGIBILITY CRITERIA

3.4.1 Inclusion criteria:

All healthy, consenting, non-pregnant (as ascertained by history) residents at least 1 year of age of the study area will be included in the trial.

3.4.2 Exclusion criteria

The following will be excluded from the trial:

- individuals who are too weak to get out of bed to receive the vaccine at the vaccination center;
- pregnant women (identified through verbal screening); and
- those less than 1 year of age.

- 1. ALLOCATION OF AGENTS

Allocation of vaccine will be done through cluster randomization. All eligible members of each cluster will receive the same agent, either vaccine or placebo. Using the data collected during the census and surveillance period, each premise will be randomly allocated for its eligible members to receive the vaccine or placebo. Potential stratification variables include:

- number of households/premise
- number of individuals / premise
- number of children under 5 years of age / premise
- mean age of eligible individuals (i.e. ≥ 1 year of age) / premise
- number of diarrhoea episodes / premise
- number of laboratory-proven cholera cases / premise
- Ward

3.6 BLINDING AND CODE BREAKING PROCEDURES

Blinding will be achieved in this trial by masking the identity of the agents using code letters for the vaccine and for the placebo. To maintain blinding, Shanta Biotechnics, will code the agents in Hyderabad prior to shipment to the study site. The consultant statistician, Prof Alan Donner, who will otherwise not be involved in this trial, will match each premise to the appropriate letter code indicating the agent to be received by all members of the premise. The sub-sample of individuals for collection of blood for serology will be randomly pre-selected by the same consultant.

The identity of the codes will only be known to IVI statistician Dr Jin Kyung Park (from Dr Allan Donner) and the Shanta staff labelling the agents. Dr Park will provide the identity of the codes (in sealed envelopes) to Shanta (Dr Raman), the DSMB, and the on-site clinical monitor (Dr Asoke Chaudhuri). Each sealed envelope will be labelled with a letter code on the outside and the identity (vaccine or placebo) on the inside.

NICED staff conducting trial and the trial participants will be blinded as to the identity of the agents. The only persons in the field site with access to the identity of the codes will be the on-site clinical monitor. He will be allowed by the DSMB to un-blind the codes in the event of severe putative vaccine reactions. Otherwise, the codes will not be revealed until the end of the trial and until the computerized dataset has been frozen. If the intervention assignment is un-blinded, the DSMB, IVI, and Shanta should be notified immediately.

- 1. DURATION OF THE STUDY PERIOD FOR ONE SUBJECT

The duration of follow-up for each subject would be 36 months after vaccination.

**4. STUDY PROCEDURES**

- 1. CENSUSES

Just prior to the phase III trial, a repeat census of the current study site and a baseline census to extend the study site to include all of Ward 29 and Ward 33 to achieve the required sample size of 110,000 subjects were conducted. During the baseline census, a unique ID number was assigned to each household member, and each household was issued an ID card. As with the baseline census, the second census registered the *de jure* population, that is the normal residents with no plans to move during the next year and who usually live in the household but may be absent during the census. A closeout census will be conducted at the conclusion of the phase III trial.

- 1. FOLLOW-UP AFTER IMMUNIZATION
     1. Follow-up for adverse events and serious adverse events

Adverse events and serious adverse events are defined under section 5.1 below. Reporting of serious adverse events is described under section 5.2 below.

Neither the bivalent, killed, oral cholera vaccine nor the placebo is known to cause any adverse reactions. However, as a precaution, procedures will be in place to detect adverse events in the trial participants as follows. All participants will be observed for 15 minutes immediately after receiving the agent. Individuals with immediate adverse events will receive emergency treatment and the event will be recorded in the Non-serious or Serious adverse event Form, as appropriate (see Appendix 4). Each group of immunization teams will be supervised by a medical officer with emergency supplies and a field car, in case of any serious adverse event requiring immediate care and transport to hospital.

The Non-serious or Serious adverse event form, as appropriate (see Appendix 4), will be completed for all adverse events that are reported within 2 weeks after each immunization whether they are considered vaccine-related or not. In addition, the CHWs will continue to visit each subject in the study site using pre-printed forms. The CHWs will inform his/her field supervisor about hospitalizations and deaths. Any hospitalization or death in a vaccinee occurring within 2 weeks after each immunization whether they are considered vaccine-related or not will be investigated immediately and reported using the Serious adverse event Form (see Appendix 4).

- - 1. Serology

After informed consent (see Appendix 2), 300 participants will be selected by stratified random sampling by the statistician based on: allocated agent (each code letter of the vaccine or placebo) and age group (less than 5 years, 5 to 15 years and over 15 years of age). These participants will be requested to provide 3 - 7 ml of venous blood at the time of the first dose, 2 weeks after the second dose, and 1 year after the first dose for testing of vibriocidal antibody. The blood samples will be kept on ice until transmittal to the laboratory (within 6 hours of collection). At the laboratory, serum will be separated and stored at -20°C until testing for antibodies. Technicians unaware of the codes of the agents received by the trial participants will test in random order paired serum samples for vibriocidal antibody titres.

Serum vibriocidal antibodies to *V. cholerae* O1 (El Tor Inaba; strain T19479) will be evaluated by a microtiter assay (14). To measure vibriocidal antibodies to *V. cholerae* O139, O139 vibrios of two different strains-the partly encapsulated vaccine strain 4260B and the capsule-deficient mutant strain M010-T4 (Virus Research Institute, Cambridge, Mass.) will be used.. The vibriocidal titre will be defined as the highest dilution causing complete inhibition of bacterial growth. Two-fold serial dilutions of pre- and post-immunization specimens will be tested side-by-side in duplicates. Titres will be adjusted in relation to a reference serum specimen included in each test to compensate for variations between analyses on different occasions. The antibody titre ascribed to each sample will be the mean of the duplicated determinations, which will not be allowed to vary more than one two-fold dilution for either the reference or the test sera. The tests will be repeated if larger variations are observed. A four-fold or greater increase in titre between pre- and post-immunization sera will be taken to represent sero-conversion.

- - 1. Surveillance for cholera

The project health outpost-based surveillance system for diarrhoea will continue after immunization. The crucial feature of the surveillance is the accurate identification of all trial participants, so that diarrhoeal outcomes can be linked to the earlier receipt of the cholera vaccine or the placebo. ID cards and computerised and printed census booklets will continue to be used to identify the individuals and their unique identification number during presentation to any of the project health outposts. Cholera-confirmed cases will continue to be visited at home 7 days after presentation to verify identification and record outcome.

Each project health outpost will be open from 8 AM to 8 PM and will continue to be staffed by a physician, field supervisor and sample collector(s) working in shifts. Those study patients who go to the IDH or B.C. Roy Children’s Hospital seeking care for diarrhoea will also be captured into the surveillance system through the outposts at these referral hospitals. The outposts at these referral hospitals will be open 24-hours a day throughout the week. Individuals in the study community will be requested to seek care for diarrhoea at any one of the project health outposts.

a. Data collection - As is being done during the pre-immunization surveillance period, for patients with diarrhoea presenting to a project health outpost, information will be obtained, a physical examination will be done, and a stool sample or rectal swab collected. Data will be entered into Case Report Form - Day 0 (CRF-0), as shown in Appendix 6. The completed forms will be sent daily to the data management room.

Treatment will be given to patients in accordance with National Guidelines. The physician and field supervisor will provide packets of Oral Rehydration Salts and provide instructions to the patients or their caretakers. In addition, the physician will refer all severe cases to the IDH or B. C. Roy Children’s Hospital. Once the laboratory results are available the study team will notify the patient and modify the treatment, as necessary.

b. Specimen collection - A stool sample or rectal swab will be obtained from each consenting patient enrolled in the surveillance for testing for *V cholerae.* The rectal swab will be put into Cary-Blair transport medium, kept in the outpost at room temperature until collected for transport to the laboratory. Each rectal swab will be labelled with the following information: patient’s name, the unique household and individual number, and date of sample collection. The Laboratory Transmittal/Result Form (see Appendix 6) will accompany the faecal specimen to the laboratory. The faecal specimen will be transported to the NICED laboratory every day within 6-8 hours of collection and processed promptly. The date and time of: (a) obtaining the specimen from the patient, (b) arrival the specimen at the laboratory, and (c) inoculation of all specimens will be properly recorded (see Laboratory Transmittal/Result form, Appendix 6).

c. Laboratory procedures for isolation of *V. cholerae -* Procedures recommended by WHO will be used to identify *V. cholerae* (15)*.* Swabs plated directly onto Eiken thiosulfate citrate bile salt sucrose (TCBS) agar as well as after enrichment in alkaline peptone water (APW) for 6 and 20 hours (pH 8.6, 37^O^C). After overnight incubation, suspected colonies from TCBS will be tested biochemically and agglutinated with polyvalent, Ogawa and Inaba antisera. Biotyping of O1 isolates will be done with chicken erythrocyte agglutination tests and with determination of polymyxin sensitivity. Non-agglutinating strains will be tested with antiserum to *V cholerae* O139 strain. *V. cholerae* isolates will be tested for susceptibility to the following antimicrobials: tetracycline, erythromycin, furazolidone, trimethoprim-sulfamethoxazole, ciprofloxacin and norfloxacin. In the laboratory, a results book will be maintained for each rectal swab received. The Laboratory Transmittal/Result Form (see Appendix 6) will be completed and sent to the data management room.

d. Follow-up visits - For those with laboratory-confirmed cholera, a follow-up visit 7 days after the initial presentation will be done to assess clinical progress and cholera-related disability (see Cholera Follow-up form, appendix 6). In case of death, a verbal autopsy will be done after an appropriate period of bereavement but within 3 months of death (see Verbal Autopsy Form, Appendix 6).

4.3. PREMATURE TERMINI NATION

Each subject or subject’s parent/guardian is free to decide whether he/she or his/her child should participate in this study. Each subject or subject’s parent/guardian will be able to withdraw him/her or his/her child from the trial at any time without resulting prejudice in health care received at any of the project health outposts.

**5. ADVERSE EVENTS**

- 1. DEFINITIONS

An adverse event following immunization is a medical incident that takes place after immunization and causes concern. Adverse events may either be causally or coincidentally related to the immunization. Possible adverse events following immunization with the killed whole-cell oral cholera vaccine include abdominal pain and cramps, loss of appetite, nausea, general ill feeling, fever and vomiting. Follow-up for adverse events following immunization will be conducted and recorded as described in section 4.2.1 above.

# Serious adverse events are those which are incapacitating, preventing normal activities, including death, life-threatening events, hospitalization, disability, and occurrence of malignancy. Follow-up for serious adverse events following immunization will be conducted and recorded as described in 4.2.1. The relationship of administration of the study agent to the serious adverse event will be assessed in each case by the clinical monitor as follows:

Not related: The serious adverse event has no relation to administration of the study agent, (i.e. existence of a clear alternative explanation, an unreasonable temporal relationship between the study agent and the event, or non-plausibility).

Unlikely related: A clinical (including a laboratory test) abnormality, with a temporal relationship to administration of the study agent but with an improbable causal relationship, and for which other drugs, chemicals, or underlying disease could provide explanations.

Possibly related: A clinical (including a laboratory test) abnormality, with a reasonable time sequence in relation to administration of the study agent, but which could also be explained by concurrent disease or other drugs or chemicals.

Probably related: A clinical event (including a test abnormality) with a reasonable time sequence in relation to administration of the study vaccine, unlikely to be attributed to concurrent disease or other drugs or chemicals.

Definitely related: A clinical event (including a test abnormality) with a clear time sequence in relation to administration of the study vaccine and likely to be attributed to the study agent.

Unknown: A clinical event (including a test abnormality) which cannot be evaluated because information is insufficient or contradictory, and which can be neither supplemented nor verified.

- 1. REPORTING OF SERIOUS ADVERSE EVENTS

SAEs will be reported promptly to the on-site clinical monitor, Dr Chaudhuri, once the principal investigator or designee determines that the event meets the protocol definition of a SAE. Dr Chaudhuri will provide an assessment of causality.

- The investigator or designee will fax the SAE report to the DSMB and to IVI (through the external monitor, Ms Eunhee Shin), and to Shanta (through the medical monitor, Dr Raman) within 24 hours of his/her becoming aware of these events.
- The SAE form will always be completed as thoroughly as possible with all available details of the event, assessment of causality, and signed by the investigator (or designee). If the investigator does not have all information regarding an SAE, he/she will not wait to receive additional information before notifying the DSMB, IVI, and Shanta.
- After the initial SAE report, the investigator is required to proactively follow each subject and provide further information to the DSMB, IVI, and Shanta on the subject’s condition. The investigator (or designee) will follow-up subjects with SAEs until the event has: resolved, subsided, stabilized, or disappeared or the event is otherwise explained, or the subject is lost to follow-up.
- The date of final disappearance of the adverse event will be documented.
- The investigator (or designee) will always provide an assessment of causality at the time of the initial report. The DSMB may request that the investigator perform or arrange for the conduct of supplemental measurements and/or evaluations to elucidate as fully as possible the nature and/or causality of the serious adverse event. The investigator is obliged to assist. If deemed necessary by one or more SAEs, the DSMB will have the authority to call a temporary moratorium on vaccination, so that the DSMB can review the adverse events data.

**6. VACCINE SUPPLIES**

6.1 STUDY AGENTS (VACCINE AND PLACEBO)

Eligible, consenting volunteers will be allocated to one of two groups, receiving one of the following:

- 1. *Bivalent oral killed cholera vaccine*: each dose of this vaccine contains

| **KILLED WHOLE-CELL ORAL CHOLERA VACCINE** | | | | |
| --- | --- | --- | --- | --- |
| **Cell numbers** | **Inactivation** | **Strain/Serotype** | **Biotype** | **Other** |
| 2.5 x 10^10^ | Heat | O1 / Inaba | Classic | Cairo 48 |
| 5.0 x 10^10^ | Formaldehyde | O1 / Inaba | El Tor | Phil 6973 |
| 2.5 x 10^10^ | Heat | O1 / Ogawa | Classic | Cairo 50 |
| 2.5 x 10^10^ | Formaldehyde | O1 / Ogawa | Classic | Cairo 50 |
| 5.0 x 10^10^ | Formaldehyde | O139 |  | 4260B |
| Total number of Vibrio cells: O1 1.25 x 10^11^ | | | | |
| Total number of Vibrio cells: O139 5 x 10^10^ | | | | |

*b. Escherichia coli* K12 strain placebo: each dose of placebo contains heat-killed *E. coli* K12 strain in an amount whose optical turbidity is identical to that for the cholera vaccine.

Each dose of vaccine or placebo is 1.5 ml. The vaccine and placebo will be dispensed in liquid form in identical vials. Prior to the trials, aliquots from the individual lots of the cholera vaccine will have undergone extensive quality control testing at VABIOTECH, the University of Gothenburg, and Shanta for sterility, detoxifying agent, thiomersal assay, immunogenicity in animals, functional residual cholera toxin activity (rabbit skin test) and LPS content by an ELISA method using polyclonal antibody. Shanta will issue the final certificate of analysis for the vaccine and placebo.

6.2 PACKAGING AND CODING

The study agents will be packed in vials.

Each vaccine vial will be coded with one of two letters (e.g. L, O) and the placebo will be coded with one of two different letters (e.g. B, W). Each subject will receive an agent coded with the same single letter for the first and second dose (i.e. L, O, B, or W). Letters with similar appearance (e.g. O and Q) will not be chosen as codes for the vaccine and placebo. Letters used in a preceding phase II trial of the vaccine in Kolkata will also be excluded. Selection of letters and coding of the vaccine and placebo will be done in Hyderabad. The staff that will be responsible for affixing letters on each vial of the agents will not be involved in any other way in the conduct of the trial nor will be present at the study site.

6.3 STORAGE CONDITIONS

The vaccine and placebo will be stored at 2 to 8°C. Vials taken to the field will be carried in vaccine boxes designed for the Expanded Programme of Immunization (EPI). Each box will have a single ice pack, and care will be taken not to freeze the vaccine. Each box will be equipped with a thermal monitor, whose measurement will be recorded upon return from the field. Vials from each lot of vaccine or placebo will be saved for future testing if necessary.

6.4 INVESTIGATIONAL PRODUCT ACCOUNTABILITY

Shanta will obtain the import license for the bulk cholera vaccine for the clinical trials and the necessary clearances from Drug Controller General of India (DCGI). The agents will be kept in a secure place. The investigator or the person in-charge of the product management will maintain records of the product delivery to the trial site, the inventory at the site, the dose(s) given to each subject, and the return of unused doses to Shanta.

6.5 ADMINISTRATION OF STUDY AGENTS

Immunization will be done through vaccine outposts and recruiting from premise to premise in the study area. After acquisition of informed consent and ascertainment of eligibility, consenting, eligible subjects will be entered into the trial. During administration of the agents, 4 vials (one of each of the 4 codes) will be at hand. The agent to be received will be determined according to the randomization list. After vigorous shaking of the vial, 1.5 ml will be extracted into a syringe and gently squirted into the mouth of the recipient, followed by water *ad libitum*. The agent will be administered with a 2-ml disposable syringe (without needle). If it is judged that a dose is not successfully ingested (e.g., regurgitated or spat out), recipients will be offered a single replacement dose, using the same procedure. Each 5-dose vial will be opened and used up according to the randomization list. At the time of the first dose, information about vaccine administration will be entered into the Vaccination Record (see Appendix 3). Fourteen days or more after the first dose, a second dose of the same code will be administered during a second vaccination round according to the same procedures. The only contraindications to the second dose will be:

- the occurrence, after the first dose, of a severe allergic reaction (generalized urticaria, wheezing, anaphylaxis);
- a diagnosis of pregnancy after the first dose.

Information during the second round will also be entered into the Vaccination Record (see Appendix 3). Syringes used for administration of the vaccine and placebo will be disposed of after each dose, to prevent inadvertent administration of contaminating amounts of non-assigned agents.

**7. DATA MANAGEMENT PROCEDURES**

Data will be double-entered into computers in a dedicated area located at NICED, using data entry programs specially created for the project by the IVI. These programs will utilize FOXPRO for Windows; all programs will incorporate range and consistency checks. Data management will include error reports, exception lists, and summary reports for each activity. The data will be automatically backed-up at systematic intervals onto external hard drives and will include an audit trail of all sequential changes. The data management system will be augmented by automatic computer virus scanning at start-up of each data entry and data management session.

All participant data will be computerized using password protection without personal identifiers. All hard copy records and backup files will be kept under lock and key at the NICED. Access to both electronic and hard copy data will be restricted to authorized study personnel only. All electronic data will be password-protected and only staff directly involved in the study will have access to this data. All data management will be undertaken without access to the vaccine code of study subjects. Following entry of the data at NICED, a copy of the data will be provided to the IVI for final edit checks, queries, and archiving.

**8. STATISTICAL CONSIDERATIONS**

- 1. ENDPOINTS

a. Efficacy will be assessed by comparing the following between the vaccine and control groups:

- Culture-proven V. cholerae O1 diarrhoea episodes severe enough to require treatment in a health care facility (primary endpoint);

- Culture-proven V. cholerae O1diarrhoea episodes associated with severe dehydration;

- Episodes of acute watery diarrhoea associated with severe dehydration;

- Episodes of acute watery diarrhoea severe enough to require treatment in a health care facility; and

- All the above endpoints stratified by age (less than 5 and over 5 years)

b. Immunogenicity will be assesses by comparing serum vibriocidal responses in the vaccine and control groups.

8.2 SAMPLE SIZE CALCULATION

a. Efficacy:

Number of participants required: As exact data is not available, the following assumptions are made for calculation of sample size required to demonstrate efficacy after 1-year of follow-up:

- Incidence = 1.2 cases/thousand population/year
- Vaccine Protective Efficacy = 50%
- Alpha = 0.05 (one-tailed)
- Power = 0.8

To measure protective efficacy of 2 complete doses, 33,000 two-dose recipients will be required per arm of the study (or 66,000 for both arms). To obtain these two-dose recipients, we will need a **geographic population of about 110,000**– i.e., 66,000 / 0.99 to exclude those less than 1 year / 0.75 for participation / 0.90 for dose 1 to dose 2 attrition / 0.9 for loss to follow-up.

Number of vaccine doses required: Since 2 doses are to be given and assuming no drop-out between dose 1 and dose 2, we would require approximately 80,000 + 16,000 (20% wastage) = **96,000** doses each of vaccine and placebo at the field site for the phase III trial.

b. Immunogenicity: The sample size is calculated with the assumptions that it is important to evaluate whether the vaccine induces acceptable serum vibriocidal responses in relation to the placebo group. Based on an immunogenicity study of the whole-cell killed oral cholera vaccine in Bangladesh (16), we make the following assumptions.

For serum vibriocidal responses, defined as >4-fold increases between baseline and post-second dose in either Inaba or Ogawa antibodies, for each age group (less than 5 years, 5 to 15 years and over 15 years of age), we assume 1) the background rate of responses in the placebo group will be 5% after the second dose and 2) the true rate of vibriocidal responses in the vaccine groups is 25%. At p <0.05 (1-tailed), 0.8 power, and a 1 to 1 allocation of vaccine and placebo, a total of 46 subjects per group would be needed. We will require approximately 50 subjects per arm per age group, for a total of 300 entered subjects.

|  | **< 5 years** | **5 to 15 years** | **Over 15 years** |
| --- | --- | --- | --- |
| **Vaccine group** | 50 | 50 | 50 |
| **Placebo group** | 50 | 50 | 50 |

- 1. ANALYSIS PLANS

The primary purpose of the analysis is to evaluate vaccine efficacy during 36 months of follow-up after receipt of two complete doses of an assigned agent. An interim analysis after 12 months will be performed in a blinded manner. Extending the analysis to 3 years will allow comparison of the duration of protection afforded by this vaccine to the Swedish vaccine that was shown to be protective for up to 3 years.

*Definition of a case:*

To be counted as a case of *V. cholerae* O1 diarrhoea, a subject must:

- present for treatment of a diarrhoea episode whose onset was 14 to 365 days after the second dose;
- describe at least three loose stools in the 24-hour period before presentation or, if one or two loose stools or if an indeterminate number of loose stools are described, must manifest at least “some” or “severe” dehydration based on WHO guidelines (17);
- describe the diarrhoea as non-bloody;
- submit a faecal specimen yielding *V. cholerae* O1; and
- have his or her identity confirmed at the time of the field check.

If there are >7 diarrhoea-free days between a first and a second episode of diarrhoea in the same individual, the second diarrhoea episode will be considered as a separate (new) diarrhoeal episode. Evaluation of vaccine efficacy thus pertains to treated episodes of cholera rather than to visits for treatment of cholera per se.

*Ascertainment of vaccination:*

Receipt of the cholera vaccine during the mass immunization will be ascertained in the computerised vaccination registry.

*Calculation of vaccine protection:*

Vaccine protection will be calculated as (1- RR_v/u_) X 100, where: RR (relative risk) = Incidence rate of cholera among age-eligible members of premises assigned the vaccine ÷ Incidence rate of cholera among age-eligible members of premises assigned the placebo.

In the **primary** analysis, the denominator in the calculation of incidence (/1000-persons/year) will consist of the number of persons living in the study area at the time of the trial who were at least 1 year of age during vaccination. Individuals will be included in the denominator until out-migration, death, or the occurrence of cholera. This analysis will compare subjects according to the study agent actually received and will include only those subjects who satisfied the inclusion/exclusion criteria, followed the protocol, and received two complete, correct doses (per protocol analysis). Cholera episodes will be retained for this analysis if they occur at least 14 days after receipt of the second dose of the study agent.

In the **secondary** analyses of the same population denominator, we will assess the efficacy of the vaccine among every subject randomized in the study (who receive the correct or incorrect study agent, one or more doses, and complete or incomplete doses) except if he/she did not receive any dose of the study agent (vaccine or placebo) or if no post randomization data was collected for this subject (intent to vaccinate analysis). Cholera cases that will be included are similar to the primary analysis but will also include cases occurring on or after the receipt of the first dose of the study agent.

In addition we will estimate the protective impact of immunization against culture-proven *V. cholerae* O139 diarrhoea episodes severe enough to require treatment in a health care facility; culture-proven *V. cholerae* associated with potentially life-threatening dehydration; episodes of acute watery diarrhoea severe enough to require treatment in a health care facility; episodes of acute watery diarrhoea irrespective of severity; and all the above endpoints stratified by age (less than 5 and over 5 years). Seroconversion in the vaccine and control groups will also be compared. Statistical methods for these secondary analyses will be the same as those used in the primary analyses.

*Interim analysis after 1 year of follow-up:*

Data will be analysed by a statistician who is otherwise not involved in the study and blinding will be maintained until the end of the 3-year follow-up.

In order to analyse the 1 year data, for a total of approximately 67,000 2-dose recipients enrolled in the study and considering a 4-5% migration rate, in order to detect 50% vaccine protective efficacy with α=0.05 (one-tailed), we need a cholera incidence of 1.2 per 1000 persons in the placebo arm. This would be equivalent to 39 cases in the placebo arm after one year of follow-up.

All culture-confirmed cholera cases will be reviewed in a blinded manner. The unblinded statistician will review the linkage of the cholera case with the vaccine file. If there are at least 39 culture-confirmed cholera cases in the placebo arm, then analysis will be performed.

*Surveillance for 3 years*

Surveillance will be continued to complete 3 years of follow-up. Blinding will be maintained until the end of the follow-up period. Analysis at 3 years of follow-up will still be performed in a blinded manner.

- 1. Potential biases

Both medical and public health personnel and study subjects will be blinded to the agent received (cholera vaccine or placebo). Hence, one would not expect information or observation bias. However, the incidence rate of other infectious diseases such typhoid fever, will also be compared between the vaccine and control groups to test similarity between the two groups with respect to health seeking behaviour. Confounding variables could also be a source of bias. Stratified randomization would try to ensure balance. Should any imbalance be raised at time of the analysis, a logistic regression model will be fitted to control for these.

**9. MONITORING, AUDITING, INSPECTION**

9.1 RESPONSIBILITIES OF THE INVESTIGATOR(S)

The principal and co-investigators at NICED will be responsible for overall supervision and management of the project. Field investigators and other co-investigators will support the PI. The PI and co-investigators will supervise all activities at health facilities and in the sites (demographic surveillance, data collection, and samples collection and transportation). They will also visit the study sites regularly. Besides other tasks, supervisors shall pay special attention to specimen collection, transportation and storage of samples in NICED, quality assurance of laboratory procedures and storage of isolated strains. The organogram is shown in Appendix 7.

The principal investigator will ensure that all Case Report Forms will be completed and computerised in real time (that is within 24 to 48 hours of completion of the form) to assure accurate and timely data. Any forms with queries or inconsistencies noted during data entry will be sent back to the field for correction or clarification. The Investigator’s Statement is shown in Appendix 1.

- 1. RESPONSIBILITIES OF THE MULTI-COUNTRY STUDY COORDINATORS

The multi-country study coordinators will ensure that the trial is adequately monitored. At regular intervals, contact with the study site will be made through visits, e-mail, and telephone calls to review the study progress and adherence to the protocol, as well as detect and correct problems. During the monitoring visits, the following will be examined: subject informed consent, subject recruitment and follow-up, vaccine allocation, vaccine storage and transport, follow-up visits, and laboratory procedures. The coordinator(s) will discuss any problems with the investigators and define, after deliberation, any action(s) to be taken.

- 1. RESPONSIBILITIES OF THE DSMB

Representatives of the DSMB will visit the study site as needed. They will be independent of and separate from the activities of the IVI staff. They will evaluate trial conduct and compliance with the protocol, the standard operating procedures, Good Clinical Practice, and the applicable regulatory requirements. This may occur at any time from start to after conclusion of the study.

**10. ETHICAL AND REGULATORY STANDARDS**

- 1. ETHICAL PRINCIPLES

The justification for using a placebo in this study is that: 1) no cholera vaccine is currently recommended for use in public health programs in India and 2) a valid assessment of vaccine efficacy in Kolkata, which is endemic for diarrhoea and for cholera, can only be obtained with use of a placebo. The placebo will consist of heat-killed bacteria (*E. coli* ), which belongs to the normal human gastro-intestinal flora and produces no adverse effects. At the end of the follow-up period, if the cholera vaccine is found to be sufficiently protective, members of premises allocated to receive the placebo will be offered full (2 doses) of the oral cholera vaccine.

- - 1. Potential benefits

During both phases of the project, the potential benefits to participants include proper treatment and referral when needed, as well as free and accurate diagnosis as to whether his/her diarrhoeal disease is due to cholera. During the vaccine trial phase, the vaccine recipients are expected to benefit from the protection offered by the vaccine against cholera. If the cholera vaccine is found to be sufficiently protective, then the placebo recipients will be offered the cholera vaccine. Vitamins or food will be offered after a blood sample is taken.

- - 1. Potential risks

The potential risk to the participants will be minimal, since there is extensive documentation of the safety of the cholera vaccine to be used and all clinical and immunization procedures (venous blood collection, rectal swab collection, and oral vaccine administration) will be performed by adequately trained and experienced personnel under regular supervision. There is a very small risk of anal/rectal area skin abrasion while taking a swab from the rectal area. Additionally, there is also a small risk associated with phlebotomy for patients who are requested to give a blood sample. This may include pain, redness and, very rarely, local infection at the phlebotomy area.

- - 1. Risk minimization

All personnel involved in taking biological samples are trained personnel, who will be provided with additional training to avoid or minimize the possibility of any unplanned side effects of these procedures. Sterile technique and disposable sterile needles and syringes will be utilized to obtain blood. All study records and data will be kept confidentially under lock and key and/or electronic password protection, as appropriate, for 10 years.  Only senior study personnel will have access to these records.

- - 1. Risk/benefit ratio

The direct benefit the participants may expect from participating in this study will be free laboratory examination and treatment for diarrhoea diseases.

The main benefit of obtaining data on the efficacy of the cholera vaccine in Kolkata will be that these data will be crucial in guiding the decision of the Indian, and perhaps other developing country governments as to whether to include the vaccine among routine public health measures against cholera. All recipients of the vaccine will potentially benefit from the probable protective effects against cholera. The risks associated with the use of the vaccine or the placebo and various other study procedures proposed to be used in this trial are expected to be minimal to nonexistent.

- 1. LAWS AND REGULATIONS

The study will be performed in accordance with the principles that govern biomedical research involving human subjects, specifically the ICMR Ethical Guidelines, the Declaration of Helsinki, and the International Conference on Harmonization’s Good Clinical Practice Guidelines to provide assurance that the rights, integrity, and confidentiality of trial subjects are protected and that reported results are credible and accurate.

10.3 INFORMED CONSENT

Community and individual informed consents will be obtained from eligible adult participants and the parents/guardians of participants aged <18 years; in addition, assent will be obtained from children aged 12-17 years of age (see Informed Consent form, Appendix 2). Consent and assent will be documented by signature or thumbprint on the appropriate forms and noted down in the Vaccination Record book (Appendix 3). The privacy and confidentiality of all data and information collected from trial participants, including those derived from clinical and biologic specimens will be ensured both during and after the conduct of the trial. Individuals will not be identified in any reports and publications based on the trial data.

10.4 INSTITUTIONAL REVIEW COMMITTEE

Before initiation of the study, the final protocol and the informed consent will require clearance Scientific Advisory Committee of NICED, Institutional Ethics Committee of NICED, Health Ministry Screening Committee of the Government of India, and the IVI Institutional Review Board. Clearance to import and use the vaccine in a clinical trial has been obtained from the Drugs Controller General of India.

**11. ADMINISTRATIVE ASPECTS**

11.1 RECORD RETENTION

The principal investigator and NICED will keep all trial documents for at least 5 years after the completion or discontinuation of the study.

11.2 PUBLICATIONS

NICED, IVI, and Shanta shall jointly own the rights to the data, clinical, and biological specimens, results and other findings resulting from this phase III trial. Parties are encouraged to publish the results of their work in a collaborative fashion for the benefit of the public while taking care to protect the intellectual property rights to proprietary discoveries. There shall be joint access of data. Guidelines for authorship of major, international, peer-reviewed journals will be used to establish authorship. Each party shall provide the others with a copy of each manuscript and abstract at least 30 days before submission for publication in a journal or presentation at an international meeting. The parties will have the right to examine the publication before it is printed and disseminated, and to request changes to the use of their name.

Subject to agreement on a case-by-case basis, each party is encouraged to produce and disseminate electronic versions of important publications produced as a result of this Cooperative Agreement. Each party will permit the others to disseminate such electronic versions as long as all original formatting, credits, and contents are maintained.

The contribution of all parties involved in this Cooperative Agreement shall be acknowledged in all abstracts, reports, or other peer-reviewed scientific publications containing data or information collected during the Project duration.

11.3 PROTOCOL AMENDMENTS

Any amendment to the protocol must be discussed and agreed upon by both the NICED investigators and IVI. If agreement is reached, such amendment will be submitted in writing for Ethics Committee approval.

An administrative change to the protocol is one that modifies administrative and logistic aspects of a protocol and that does not affect the study participants’ safety, the objectives of the trial and its progress. An administrative change may require Ethics Committee modification.

The principal investigator is responsible for insuring that changes in the trial may not be initiated without Ethics Committee review and approval, except when necessary to eliminate immediate hazards to human subjects.

**12. REFERENCES**

1. Government of India. Health Information of India 1995, DGHS, New Delhi.
2. Sur D, Dutta P, Nair GB, Bhattacharya SK. Severe cholera outbreak following floods in a northern district of West Bengal. *Indian J Med Res* 2000; 112: 178-82.
3. Radhakutty G, Sircar BK, Mondal SK, Mukhopadhyay AK, Mitra RK, Basu A, Ichpugani I, Nair GB, Bhattacharya SK. Investigation of an outbreak of cholera in Allepey and Palghat districts, South India. *Indian J Med Res* 1997; 106: 455-7.
4. Niyogi SK, Mondal S, Sarkar BL, Garg S, Banerjee D, Dey GN. Outbreak of cholera due to *V cholerae* O1 in Orissa state. *Indian J Med Res* 1994; 100: 217-8.
5. Ramarmurthy P, Garg S, Sharma R, Bhattacharya SK, Nair GB, Shimada T,, Karasawa T, Kurazono H, Pal A, Takeda Y. Emergence of a novel strain of *Vibrio cholerae* with epidemic potential in southern and eastern India. *The Lancet* 1993; 341: 703-4.
6. World Health Organization Expert Committee on Biological Standardization. Guidelines for the production and control of inactivated oral cholera vaccines. 26 – 30 November 2001, Geneva. BS/01/1938.
7. World Health Organization Global Task Force on Cholera Control. Cholera Vaccines: A new public health tool? Report of a WHO meeting, 10-11 December 2002, Geneva, Switzerland. WHO/CDS/CPE/ZFK/2004.5.
8. Clemens J D et al , Field trials of cholera vaccines in Bangladesh: results from a three year follow- up. *The Lancet* 1990; 335: 270 –3.
9. Sanchez J L, Vasquez B, Beque R et al, Protective efficacy of the oral whole cell / recombinant B subunit cholera vaccine in Peruvian military recruits. *The Lancet* 1994; 344: 1273 – 6.
10. Trach DD. The problem of cholera immunization in Vietnam. *In:* Prospects for public health benefits in developing countries from new vaccines against enteric infections. Stockholm: SAREC Documentation, 1990.
11. [Trach DD, Clemens JD, Ke NT, Thuy HT, Son ND, Canh DG, Hang PV, Rao MR.](http://www.ncbi.nlm.nih.gov:80/entrez/query.fcgi?cmd=Retrieve&db=PubMed&list_uids=9014909&dopt=Abstract) Field trial of a locally produced, killed,oral cholera vaccine in Vietnam. *The* *Lancet* 1997; 349, 231-5.
12. [Trach DD, Cam PD, Ke NT, Rao MR, Dinh D, Hang PV, Hung NV, Canh DG, Thiem VD, Naficy A, Ivanoff B, Svennerholm AM, Holmgren J, Clemens JD.](http://www.ncbi.nlm.nih.gov:80/entrez/query.fcgi?cmd=Retrieve&db=PubMed&list_uids=11884967&dopt=Abstract) Investigations into the safety and immunogenicity of a killed oral cholera vaccine developed in Viet Nam. *Bulletin of the World Health Organization* 2002; 8, 2-8.
13. Sur D, Deen J, Manna B, et al. The burden of cholera in the slums of Kolkata, india: Data from a prospective, community-based study. *Archives of Diseases in Children* 2005. In press.
14. Jertborn M, Svennerholm A-M, Holmgren J. Saliva, breast milk, and serum antibody responses as indirect measures of intestinal immunity after oral vaccination or natural disease. *Journal of Clinical Microbiology* 1986; 24: 203-9.
15. Laboratory methods for diagnosis of epidemic dysentery and cholera. WHO/CDS/CSR/EDC.99.8, Centers for Disease Control, Atlanta, Georgia, 1999.
16. Clemens JD, Stanton BF, Chakraborty J, et al. B Subunit-whole cell and whole-cell only oral vaccines against cholera: Studies on reactogenicity and immunogenicity. *JID* 1987; 155:79-85.
17. World Health Organization Division of Diarrhoeal and Acute Respiratory Disease Control. The treatment of diarrhoea: A manual for physicians and other senior health workers 1995. World Health Organization, Geneva. WHO/CDR/95.3.
18. DD Anh, VD Thiem, DG Canh, AL Lopez, PT Long. Safety and immunogenicity of a reformulated Vietnamese bivalent killed, whole-cell, oral cholera vaccine in adults. Presented as a poster in the 40^th^ US-Japan Cholera and other bacterial enteric infections joint panel meeting 30 November -2 December 2005.
19. Safety and immunogenicity trials of a killed, oral cholera vaccine in Indian subjects in eastern Kolkata, West Bengal. Results of preliminary analysis.
